# Society Meetings

**Published:** 1913-11

**Authors:** 


### SOCIETY MEETINGS.

---

*Brief reports and announcements of meetings of societies of Western New York and of those of general scope are requested from Secretaries. Copy should be on hand by the fifteenth of each month. Full transactions will be published at cost of composition.*

---

The Wyoming County Medical Society held its annual meeting in Warsaw, October 14. Dr. Roswell Park of Buffalo gave a paper on "Radium and Radio-activity," and Dr. De Witt H. Sherman of Buffalo led a discussion on the use of drugs in obstetrics. Officers were elected as follows: President, W. J. French of Pike; Vice-President, W. R. Thomson of Warsaw; Secretary-Treasurer, L. H. Humphrey of Silver Springs. The January meeting will be held with Dr. Mary T. Green as hostess at the Castile Sanitarium.

At the meeting of the 8th District Branch of the Medical Society of the State of New York, held at Sonyea, September 24 and 25, in connection with the 7th District Branch, the following officers were elected: President, Dr. Arthur G. Bennett, Buffalo; 1st Vice-President, Dr. Carl Leo-Wolf, Niagara Falls; 2d Vice-President, Dr. Albert Lytle, Buffalo; Secretary, Dr. E. A. Sharp, Buffalo; Treasurer, Dr. F. H. VanOrsdale, Belmont, N. Y.

---

The Elmira Academy of Medicine held a luncheon before its regular meeting, October 24. Dr. Howard A. Kelly of Baltimore was the guest of honor, speaking on Radium.

---

The Aesculapian Club of Buffalo held a regular dinner-meeting at the Buffalo Club, October 16. Major Wm. G. Bissell, M. D., was the host. A paper on "The Other Lesion" was read by Dr. A. L. Benedict.

---

The Chautauqua County Medical Society held its triennial meeting at Jamestown, September 20. Dr. Paul B. Brooks of

Norwich read a paper on the prevention of venereal disease, and Dr. V. D. Bozovsky of Dunkirk one on the treatment of injuries of the hand. Dr. A. M. Grover and Dr. A. F. Jaeckle of Dunkirk were admitted to membership.

---

The Medical Society of the County of Erie held its regular fall meeting, October 20, in Alumni Hall, University of Buffalo. Dr. Edith R. Hatch presented a paper, "Is the Teaching of Sex Hygiene Advisable in Our Public Schools?" Drs. Lucien Howe and F. Parke Lewis led the discussion. A collation was served.

---

The Rochester Academy of Medicine, Section II, Surgery, met October 8, at the Academy Rooms. Dr. Julius Auerbach of New York presented a paper, "Chronic Middle Ear Suppuration From the Standpoint of the General Practitioner." A subscription dinner was given at the Hotel Seneca.

---

The Buffalo Academy of Medicine has held the following meetings up to date of going to press:

October 7—Surgery. Demonstration of Pathoplasts and Autochrom Photographs (of venereal lesions), Dr. Charles Bethune; Radio-activity and Radium by Dr. Roswell Park.

October 14—Medicine. Symposium on the Proposed Ordinance for the Suppression of Unnecessary Noises. Health Commissioner Fronczak's review of the subject is promised for our next issue.

October 21—Obstetrics and Gynæcology. Presentation of a case of Pseudohermaphroditism by Dr. James E. King; Buried and Subcuticular Sutures in Perineorrhaphy (illustrated by lantern slides) by Dr. Robert L. Dickinson of Brooklyn. The discussion on the latter paper was opened by Dr. Earl P. Lothrop.

---

Dr. Carl G. Leo-Wolf of Niagara Falls was the guest of the evening at a farewell dinner given by the Niagara Falls Academy of Medicine on September 25th.

On the following Tuesday, Dr. Leo-Wolf sailed for Germany, where for four months he will be the guest of Prof. Finkelstein at Berlin. The exceptional opportunities for pediatrical study at the Finkelstein Clinic will be at Dr. Leo-Wolf's command. Before returning home he will spend two months at Vienna.

